# Metagenomic insights into host-specific gastroenteritis bacteria in forest rodents of Sarawak, Borneo: implications for one health surveillance of rodent-borne pathogens

**DOI:** 10.1186/s12866-025-04241-8

**Published:** 2025-08-23

**Authors:** Muhammad Amin Iman Azmi, Julius William-Dee, Muhd Amsyari Morni, Nur Afiqah Aqilah Azhar, Nor Al-Shuhadah Sabarudin, Emy Ritta Jinggong, Syamzuraini Zolkapley, Nur Iylia Maisarah Baharom, Muhammad Danish Haqeem, Victor Lee Sien, Asfa Hanis Mohamad Azmi, Madinah Adrus, Cheng-Siang Tan, Faisal Ali Anwarali Khan

**Affiliations:** 1 Faculty of Resource Science and Technology, Universiti Malaysia Sarawak, Sarawak, Kota Samarahan 94300 Malaysia; 2 Institute of Biodiversity and Environmental Conservation, Universiti Malaysia Sarawak, Sarawak, Kota Samarahan 94300 Malaysia; 3 Faculty of Medicine and Health Sciences, Universiti Malaysia Sarawak, Sarawak, Kota Samarahan 94300 Malaysia

**Keywords:** 16S, Gut microbiota, Malaysia, Muridae, Nanopore sequencing

## Abstract

Rodents are known to act as reservoirs for zoonotic diseases due to their widespread distribution and synanthropic nature. Among these, foodborne pathogens capable of causing gastroenteritis in humans are of particular concern, as rodents can facilitate their transmission through contamination of food sources. Forest-dwelling rodents may harbour bacterial taxa native to their habitat, posing a risk of zoonotic spillover to nearby human settlements. This risk is further heightened by the growing prevalence of ecotourism activities, particularly in tropical countries like Malaysia. In this study, rodent trapping was conducted in selected forested areas adjacent to human settlements across Sarawak. Nanopore sequencing of the full-length 16 S rRNA gene was conducted on faecal DNA from 46 rodent individuals representing seven different species (*Maxomys surifer*, *M. tajuddinii*, *M. whiteheadi*, *Niviventer cremoriventer*, *Rattus tanezumi*, *R. tiomanicus* and *Sundamys muelleri*). A total of ten bacterial species associated with gastroenteritis were successfully detected. Notable findings in this study include *Campylobacter*, *Salmonella* and *Shigella* species, which are known to cause bacterial gastroenteritis in humans. Also, the presence of certain bacterial taxa in specific rodent genera suggests potential host specificity of foodborne pathogens. This study highlights the potential public health risks caused by rodent-borne bacterial transmission and the importance of monitoring forest rodents as potential reservoirs of zoonotic pathogens.

**Supplementary Information:**

The online version contains supplementary material available at 10.1186/s12866-025-04241-8.

## Background

Rodents are widely considered as reservoir hosts for zoonotic diseases as they can be carriers for diverse pathogenic bacteria [1]. Their synanthropic nature enables them to coexist near human settlements, adapting to anthropogenic environments [2]. This proximity increases the likelihood of interactions between rodents and humans, creating opportunities for disease transmission. Pathogens can be transmitted directly to humans via bites or excretions such as saliva and faeces, or indirectly through other animals, including arthropods or livestock, that come into contact with rodents [3]. Among these pathogens, bacteria that are known to cause gastroenteritis are of particular concern due to their potential to cause widespread foodborne illness in human populations.

Bacterial gastroenteritis, caused by foodborne pathogenic bacteria, is a major public health concern worldwide. Recent outbreaks of enteric bacteria in Borneo include a cholera outbreak in Kudat, Sabah, and invasive *Salmonella* infections in Bintulu, Sarawak, both occurring in urban settings [4, 5]. These bacteria can lead to gastrointestinal infections in humans, typically through the ingestion of contaminated food or water, causing a wide range of symptoms relating to diarrhoea and vomiting [6]. Rodents can serve as reservoirs for these pathogens, facilitating transmission to humans through contamination of food sources [7–9]. Detecting these pathogens in rodents is essential for understanding their role as vectors and preventing outbreaks, particularly in areas with frequent human-rodent interactions.

Previous studies in Sarawak have utilised targeted amplicon sequencing methods to detect specific strains of pathogenic bacteria in rodents [10–16]. However, recent advancements in next-generation sequencing (NGS) technologies have revolutionised unbiased amplicon sequencing methods, allowing the characterisation of complex mammalian microbiomes [17–22]. Nanopore sequencing in particular is a portable and real-time NGS technology developed by Oxford Nanopore Technologies (ONT) that produces long DNA sequence reads, enabling rapid and accurate identification of bacterial species [23]. This technology enables the comprehensive study of complete microbial communities in organisms like rodents, thereby facilitating the identification of certain bacteria that may otherwise go undetected using traditional methods [24]. These advancements in sequencing technology provide new possibilities for investigating the rodent microbiome, particularly in relation to zoonotic pathogens.

Characterising the gut microbiome allows the understanding of rodents’ normal gut microbiota and subsequently identify the carriage of potentially infectious bacteria to humans and other animals. Forest-dwelling rodents may harbour diverse gut microbiota due to their varied diets and exposure to a broader range of environmental microbes [25]. This diversity may also provide opportunities to detect pathogenic bacteria endemic to their habitats. Additionally, forested areas support a higher diversity of rodent species [26], each of which could potentially harbour host-specific bacteria. Notably [16], provided new geographical evidence of novel pathogenic bacteria in rodents from forested areas in Sarawak. Their findings highlight the significance of boundaries between forest fringes and human settlements, which may serve as interfaces for the transmission of zoonotic diseases [1, 27].

Furthermore, ecotourism is rapidly expanding in tropical forest regions, including Malaysia, due to growing interest in nature-based tourism activities [28]. While ecotourism supports sustainable economic development and promotes biodiversity conservation, the accompanying rise in human activity may disrupt natural habitats and alter wildlife behaviour [29, 30]. As a result, wild rodents may be displaced toward nearby human settlements, increasing opportunities for zoonotic spillover. These interfaces highlight the importance of a One Health approach, which recognises the interconnected risk of disease transmission across humans, wildlife and environmental health. Therefore, the main objective of this study is to determine the distribution of bacterial pathogens associated with gastroenteritis across rodent species in selected undisturbed forested regions of Sarawak, providing a baseline for assessing potential disease transmission risks in the context of ecotourism and human encroachment.

## Methods

### Study sites

**Fig. 1:**
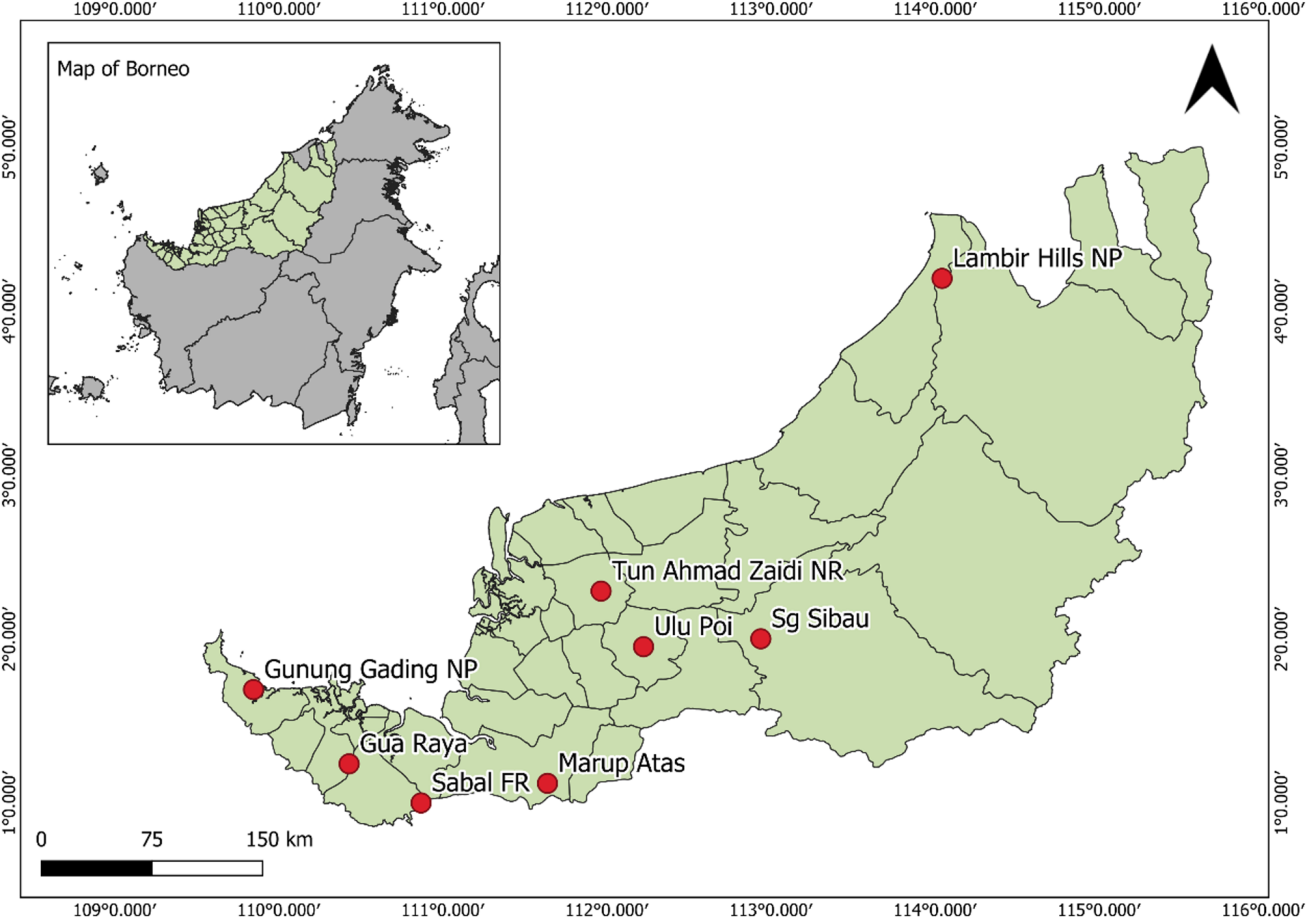
Map of sampling sites indicated by red dots in Sarawak, Borneo

The study sites consist of several forested areas throughout Sarawak that have minimal anthropogenic pressure, maximising the likelihood of recovering bacteria native to the region. At the same time, their proximity to human settlements and the prevalence of ecotourism at some of these sites provide an opportunity to investigate potential zoonotic spillover. These sites (Fig. 1) include totally protected areas, such as Gunung Gading National Park (N1°41’27.0” E109°50’45.0”), Lambir Hills National Park (N4°11’57.5” E114°02’34.3”), Sabal Forest Reserve (N0°59’59.1” E110°52’2.1”) and Tun Ahmad Zaidi Nature Reserve (N2°17’29.9” E111°57’50.96”), as well as non-protected areas including Marup Atas Engkilili (N1°07’08.3” E111°38’15.8”), Sungai Sibau Kapit (N2°00’02.7” E112°56’16.4”), Ulu Poi Kanowit (N1°57’09.9” E112°13’23.0”) and Gua Raya Serian (N1°14’18.0” E110°25’46.8”). Rodent trapping was conducted between October 2021 and December 2024. On average, trapping was conducted over five sampling nights per site, with each site sampled at different time periods (Supplementary Table 1).

### Sampling methods

Wild forest rodents were caught in accordance with permits issued by the Sarawak Forestry Corporation (Permit No. WL21/2021 and 040/2024). On average, a total of 100 Sherman traps and 100 cage traps were used for rodent trapping for each study site. The traps were sterilised with 70% ethanol and bleach before being baited with banana coated in peanut butter. Traps were checked daily from 0900 h to 1200 h and re-baited when necessary. To minimise cross-contamination of samples, captured rodents were transferred to separate sterilised, breathable containers for faecal sample collection (multiple pellets, each 1–2 cubic mm). Morphological measurements (sex, weight, ear, hindfoot, tail ventral, and head-body length) of the captured rodents were also recorded and identified based on descriptions and keys from [31] and [32]. Fresh faecal samples from the captured individuals were preserved in RNAlater (Ambion, USA) and kept in −20 °C conditions for long term storage. The rodents were then released back into their respective habitats following faecal sample collection.

### Next-generation sequencing

DNA was extracted from the collected faecal samples using the QIAamp PowerFecal Pro DNA Kit (Qiagen, Hilden, Germany), following the manufacturer’s protocol. The extracted faecal DNA was then quantified using a DeNovix DS-11 Spectrophotometer (Denovix, Delaware, USA). Samples passing the quality check (purity ratio between 1.8 and 2.0 at 260/280 nm absorbance; DNA concentration above 20 ng/µl) proceeded to nanopore sequencing. The 16 S Barcoding Kit (SQK-RAB204; SQK16S114.24) from Oxford Nanopore Technologies (ONT, Oxford, United Kingdom) was used according to the manufacturer’s protocol to amplify the 16 S rRNA gene with the universal primers, 27 F and 1482R, included in the kits. The same barcoding kit was also used for sample pooling and library preparation. The R9.4.1 flow cells were used for the older sequencing kits (SQK-RAB204) while the R10.4.1 flow cells were used for the newer kits (SQK16S114.24). Finally, nanopore sequencing was carried out using a MinION Mk1C sequencer (ONT), with each sequencing run lasting approximately 17 h.

### Data analysis

FASTQ files of the 16S rRNA sequences were basecalled using Dorado 7.6.7 (ONT), integrated within the MinKNOW 24.11.8 (ONT) software. Reads below the quality score of 8 were filtered out of the dataset. Taxonomic classification of bacteria was performed up to the species level using Emu v3.5.1, which employs an expectation-maximization algorithm for long-read sequences [33]. Emu reduces false positives and enables accurate species-level classification by accounting for high error rates and extended read lengths [33]. The default Emu reference database used was a combination of NCBI 16 S RefSeq [34] and rrnDB v5.6 [35]. The R package *phyloseq* [36] was used to construct operational taxonomic unit (OTU) tables and taxonomy tables. The dataset was then normalised to the sample with the lowest read count (~ 25,000 reads) using rarefaction in *phyloseq*.

To examine relevant bacterial taxa, the dataset was further filtered based on genera and/or species known to cause bacterial gastroenteritis [6, 37, 38]. A heatmap of log-transformed bacterial abundance was generated using the *ggplot2* package [39] to visualise the distribution of these bacteria across rodent hosts. For overall bacterial composition analysis, a non-metric multidimensional scaling (NMDS) plot was constructed using the full, unfiltered dataset to represent the beta diversity of microbial communities among rodent genera. The NMDS analysis was performed using the *ggplot2* package, based on Jaccard [40] and Bray-Curtis [41] indices. Lastly, a permutational multivariate analysis of variance (PERMANOVA) test was conducted for both indices to assess significant differences in bacterial composition between rodent genera.

## Results

**Fig. 2:**
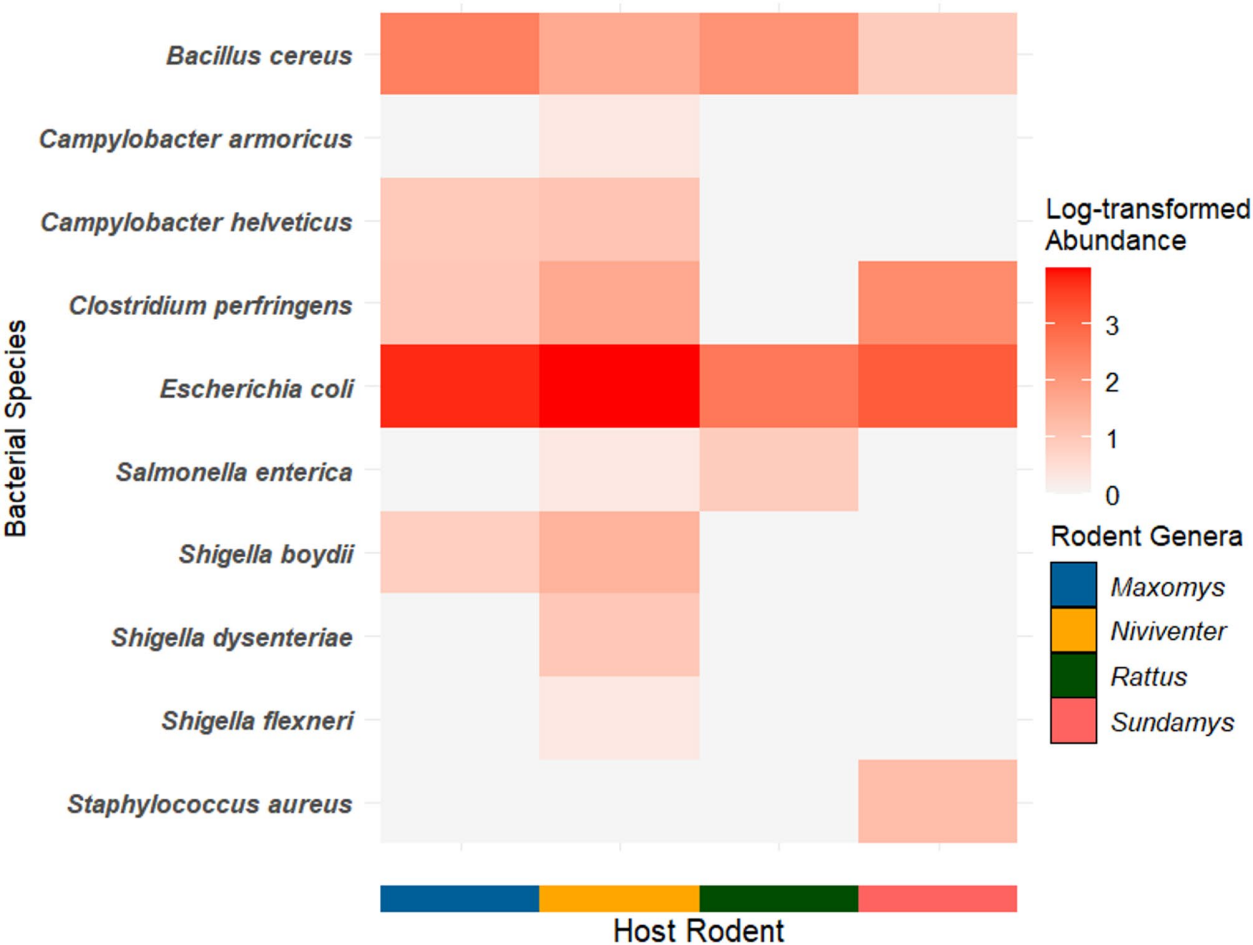
Heatmap showing the log-transformed abundance of bacterial species associated with gastroenteritis across different rodent genera

A total of 46 individuals of rodents, representing seven different species, *Maxomys surifer* (*n* = 1), *M. tajuddinii* (*n* = 5), *M. whiteheadi* (*n* = 16), *Niviventer cremoriventer* (*n* = 12), *Rattus tanezumi* (*n* = 2), *R. tiomanicus* (*n* = 2) and *Sundamys muelleri* (*n* = 8), were captured and included in this study (Supplementary Table 1). Among the species recorded, *Rattus tanezumi* is likely an introduced species in Borneo [42], while the remaining species are indigenous to the Southeast Asian region [43]. After filtering for bacterial taxa associated with gastroenteritis, ten bacterial species were detected in the sampled rodents. Based on the heatmap of bacterial species detected in rodent genera (Fig. 2), bacterial species such as *Bacillus cereus* and *Escherichia coli*. were present across all four rodent genera (*Maxomys*, *Niviventer*, *Rattus* and *Sundamys*). However, some bacterial species were only detected in specific rodent hosts. For instance, *Campylobacter armoricus*, *Shigella dysenteriae* and *S. flexneri* were found exclusively in *Niviventer* individuals. A more detailed heatmap, displaying bacterial presence at the individual rodent sample level, is available in Supplementary Fig. 1.

**Fig. 3:**
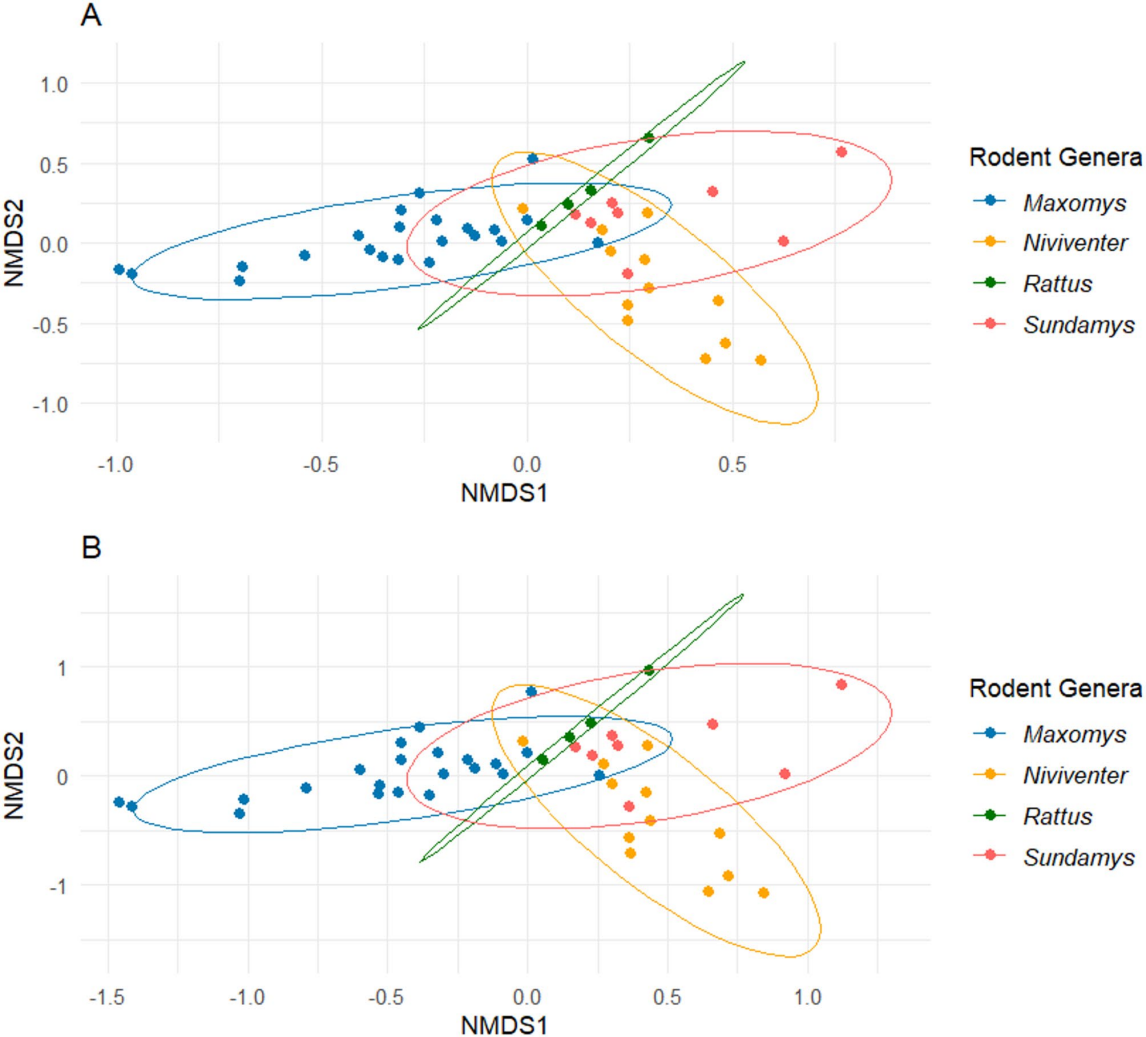
Non-metric multidimensional scaling (NMDS) plots based on (**A**) Jaccard and (**B**) Bray-Curtis indices. Colours represent different rodent genera

Beta diversity analysis revealed a significant overlap in microbial composition across all rodent genera (Fig. 3). However, *Maxomys*, *Niviventer* and *Sundamys* formed more distinct clusters, indicating variation in bacterial community structures. These differences were statistically supported, with significant variation in bacterial compositions among rodent genera based on both the Jaccard (PERMANOVA: R² = 0.1739; *p* < 0.001) and Bray-Curtis (PERMANOVA: R² = 0.2379; *p* < 0.001) indices. Pairwise comparisons further revealed that *Maxomys* and *Niviventer* were significantly different from all other genera, with at least *p* < 0.05 for all comparisons (Table 1). In contrast, *Sundamys* and *Rattus* were not significantly different with each other (Jaccard: *p* = 0.325, Bray-Curtis: *p* = 0.240), supporting their observed overlap in bacterial composition.

**Table 1:** Pairwise permutational multivariate analysis of variance (PERMANOVA) comparisons of bacterial compositions between rodent genera based on Jaccard and Bray-Curtis indices. * = *p* ≤ 0.05, ** = *p* ≤ 0.01, *** = *p* ≤ 0.001

Index		Maxomys vs. Niviventer	Maxomys vs. Sundamys	Maxomys vs. Rattus	Niviventer vs. Sundamys	Niviventer vs. Rattus	Sundamys vs. Rattus
Jaccard	*R*^2^ value	0.1326	0.1076	0.0742	0.1113	0.1231	0.0965
	*p* value	0.001***	0.001***	0.011*	0.018*	0.029*	0.325
Bray-Curtis	*R*^2^ value	0.1941	0.1574	0.0972	0.1458	0.1579	0.1034
	*p* value	0.001***	0.001***	0.010**	0.014*	0.018*	0.240

## Discussion

This study provides novel insights into the role of wild rodents as reservoirs for foodborne pathogens. The presence of these bacteria in rodents from forested areas, distant from human settlements, suggests that they may be naturally occurring in these environments rather than exclusively linked to anthropogenic activity. To our knowledge, this study is the first to report the detection of *Campylobacter* and *Shigella* in wild rodents in Malaysia. These findings highlight the need for disease surveillance in minimally disturbed habitats, where the occurrence of native microbes may differ from those commonly detected in urban or agricultural landscapes [16].

Certain bacterial genera associated with gastroenteritis, such as *Yersinia* and *Vibrio*, were not detected in this study. Their absence may be attributed to environmental conditions at the study sites or host specificity, as these bacteria might not typically associate with the rodent species examined [44, 45]. Additionally, the study focused on bacterial taxa with well-documented associations with gastroenteritis, which may have led to the exclusion of other potentially relevant species. Nevertheless, the identified taxa reinforce the role of wild rodents as reservoirs for foodborne pathogens.

ESKAPE pathogens, including *Escherichia coli* and *Staphylococcus aureus*, were detected in this study, both of which are known to acquire antimicrobial resistance [46]. While these species are included in the World Health Organization (WHO) priority pathogen list [47], their presence alone does not indicate pathogenicity, as both species can exist as commensal bacteria within humans [48, 49]. This study identified these species using full-length 16 S sequencing, which does not distinguish between commensal and pathogenic strains. Determining their pathogenic potential would require additional analyses, such as detecting virulence-associated genes or conducting antibiotic susceptibility testing [12, 50].

Among the prevalent bacterial taxa detected in this study, the genus *Shigella* is a well-known cause of bacterial gastroenteritis. *Shigella* species are the main causative agents of shigellosis, a disease characterised by abdominal pain, watery diarrhoea and dysentery, which presents as scant stools tinged with mucus and blood [51]. Notably, three recognised *Shigella* species (*S. boydii*,* S. dysenteriae* and *S. flexneri*,) were identified in this study, with their presence detected in *Maxomys* and *Niviventer* individuals. *Shigella* is known to be highly transmissible, with as few as 10–100 organisms capable of causing infection in humans [51]. Also, the emergence of antimicrobial-resistant strains of this genus in Malaysia warrants further surveillance and research of such strains [52]. The detection of *Shigella* in rodents from forested environments suggests that wild rodents may serve as important reservoirs for this bacterium in natural ecosystems, highlighting the potential role of wildlife in the transmission of foodborne pathogens.

Another notable finding is the detection of *Salmonella enterica* in *Niviventer*, and *Rattus* rodents. Non-typhoidal strains of *S. enterica* are commonly associated with salmonellosis, a foodborne illness that can lead to gastrointestinal symptoms such as diarrhoea, fever and abdominal cramps, with severe cases resulting in systemic infections [53]. Previous studies on the prevalence of *Salmonella* in Malaysia have primarily focused on urban environments and poultry farms, where the risks of spillover to human settlements and food sources are more apparent [54–56]. However, the detection of *Salmonella* in rodents from forested habitats suggests that these bacteria persist across diverse environmental conditions, highlighting their widespread distribution beyond anthropogenic landscapes.

Furthermore, *Campylobacter* was detected exclusively in *Maxomys* and *Niviventer* as *C. helveticus* was present in both rodent genera, while *C. armoricus* was found only in *Niviventer*. Notably, *C. jejuni*, one of the most common causes of bacterial gastroenteritis worldwide [57], was not detected in this study. The absence of *C. jejuni* in this study could be attributed to forest-dwelling rodents not being natural hosts, as this bacterium is more commonly associated with animals from agricultural landscapes [7]. The presence of relatively uncommon *Campylobacter* species in this study highlights the role of forest rodents as reservoirs for microbes native to their surrounding habitat.

The detection of *Campylobacter* and *Shigella* in only *Maxomys* and *Niviventer* suggests potential host specificity, which may be influenced by ecological factors. The presence of these bacteria in *Maxomys* and *Niviventer*, but not in *Rattus* and *Sundamys*, could be linked to habitat preferences. In Malaysian Borneo, *Maxomys* and *Niviventer* are typically restricted to primary and secondary forests [26, 31]. In contrast, *Rattus* species are commonly found in urbanised landscapes and plantations, while *Sundamys muelleri* (the only *Sundamys* species sampled in this study) inhabits both undisturbed forests and those experiencing anthropogenic pressures, such as forest fringes and recreational forests [26, 31, 58]. These habitat differences may shape the overall gut microbial composition of these rodents, as reflected in the beta diversity analysis of this study. However, the specific ecological factors that could have influenced their gut microbiota remain unclear. Investigating host genetics, dietary preferences of rodents and forest vegetation structure could potentially reveal these associations [59–61].

While the use of next-generation sequencing has allowed for the detection of bacteria associated with gastroenteritis within rodent populations, some challenges were faced due to the disparity in library sizes between samples. Due to the nature of high throughput sequencing, the number of reads and sequencing depth differ across samples, resulting in uneven datasets. To improve this, the rarefying method was used to eliminate bias associated with differences in sample size by subsampling each sample set to a shared threshold. Although this may have resulted in the underestimation of rare taxa, a large, normalised library size of approximately 25,000 reads per sample was retained as shown to minimise the introduction of artificial variation [62].

Additionally, only full-length 16 S rRNA gene sequencing was performed to detect bacteria commonly associated with gastroenteritis. The detection of these bacteria does not confirm their pathogenicity or potential to cause diseases in humans. To determine pathogenicity, bacterial culturing and antibiotic susceptibility testing should be conducted [63]. Furthermore, future studies should investigate pathogenicity islands, which are genomic regions that encode virulence factors and contribute to bacterial pathogenicity [64]. Nevertheless, the bacteria identified in this study such as *Campylobacter*, *Salmonella* and *Shigella* are primarily pathogenic and are not recognised as commensal bacteria in the human gut [6]. Infection in humans would likely cause gastroenteritis symptoms, further reinforcing the role of forest rodents as potential reservoirs for these pathogens.

## Conclusion

This study successfully detected gastroenteritis-associated bacteria in rodents from forested areas in Sarawak. The presence of *Campylobacter*, *Salmonella* and *Shigella* in rodents from minimally disturbed habitats highlights the potential for these bacteria to occur beyond anthropogenic landscapes, suggesting that they may persist in natural ecosystems independent of direct human activity. Given their potential to cause gastrointestinal infections, habitat disturbances caused by increased human-wildlife interactions through ecotourism could increase the likelihood of pathogen spillover from wildlife to human populations. Additionally, the restricted distribution of *Campylobacter* and *Shigella* species among specific rodent genera suggests potential host specificity, which may be influenced by ecological factors such as host genetics or habitat preference. However, these observations should be interpreted cautiously due to the limited sample size. Similarly, the apparent absence of other enteric bacteria may be attributed to sampling limitations rather than true absence. While next-generation sequencing enabled broad bacterial detection, it could not distinguish between pathogenic and commensal strains, necessitating further investigations to determine pathogenic potential. Overall, this study underscores the importance of adopting a One Health approach to monitor zoonotic pathogens in natural ecosystems, highlighting the need for integrated surveillance that considers wildlife, environmental and public health sectors.

## Supplementary Information

Supplementary Material 1.

## Data Availability

The raw FASTQ sequences used for this study are available in the BioProject database under the accession numbers PRJNA1023681 and PRJNA1257425. The specific BioSample accession numbers for each individual sample are available in Additional file 1: Supplementary Table 1.
