# Supplementary material for: Metagenomic insights into host-specific gastroenteritis bacteria in forest rodents of Sarawak, Borneo: implications for one health surveillance of rodent-borne pathogens
Source: BMC Microbiol. 2025 Aug 23;25:531. doi: 10.1186/s12866-025-04241-8 (PMC12374452; doi:10.1186/s12866-025-04241-8)

**Supplementary Table 1.** Sample information of rodents used in this study.

|  |  |  |  |  |  |  |  |  |  |  |  |  |
| --- | --- | --- | --- | --- | --- | --- | --- | --- | --- | --- | --- | --- |
| **Sample ID** | **Field ID** | **Species** | **Age** | **Sex** | **E (mm)** | **HF (mm)** | **TV (mm)** | **HB (mm)** | **WT**  **(g)** | **Sampling Date** | **Locality** | **BioSample No.** |
| Msuri01 | SSK22-013 | *Maxomys surifer* | A | M | 21 | 42 | 200 | 180 | 150 | 16/07/2022 | Sungai Sibau, Kapit | SAMN37668518 |
| Mtaju01 | MAE22-006 | *Maxomys tajuddinii* | A | M | 15 | 29 | 125 | 118 | 60 | 20/05/2022 | Marup Atas, Engkilili | SAMN37668513 |
| Mtaju02 | MAE22-010 | *Maxomys tajuddinii* | A | M | 16 | 30 | 119 | 110 | 55 | 20/05/2022 | Marup Atas, Engkilili | SAMN37668515 |
| Mtaju03 | SSK22-009 | *Maxomys tajuddinii* | A | M | 16 | 30 | 127 | 128 | 56 | 16/07/2022 | Sungai Sibau, Kapit | SAMN37668517 |
| Mtaju04 | SSK22-012 | *Maxomys tajuddinii* | A | M | 16 | 31 | 131 | 130 | 47 | 16/07/2022 | Sungai Sibau, Kapit | SAMN48267155 |
| Mtaju05 | UPK23-005 | *Maxomys tajuddinii* | A | M | 14 | 28 | 110 | 105 | 65 | 12/03/2023 | Ulu Poi, Kanowit | SAMN37668521 |
| Mwhit01 | GGNP21-008 | *Maxomys whiteheadi* | A | M | 19 | 28 | 119 | 135 | 63 | 22/10/2021 | Gunung Gading National Park | SAMN37668508 |
| Mwhit02 | GGNP21-011 | *Maxomys whiteheadi* | A | F | 19 | 26 | 123 | 121 | 80 | 22/10/2021 | Gunung Gading National Park | SAMN37668509 |
| Mwhit03 | GGNP21-013 | *Maxomys whiteheadi* | A | M | 16 | 22 | 96 | 102 | 36 | 22/10/2021 | Gunung Gading National Park | SAMN48267156 |
| Mwhit04 | GGNP21-019 | *Maxomys whiteheadi* | A | M | 17 | 31 | 131 | 147 | 70 | 23/10/2021 | Gunung Gading National Park | SAMN48267157 |
| Mwhit05 | GGNP21-020 | *Maxomys whiteheadi* | A | M | 13 | 27 | 123 | 113 | 65 | 23/10/2021 | Gunung Gading National Park | SAMN48267158 |
| Mwhit06 | GGNP21-021 | *Maxomys whiteheadi* | A | M | 15 | 28 | 115 | 122 | 65 | 23/10/2021 | Gunung Gading National Park | SAMN48267159 |
| Mwhit07 | MAE22-009 | *Maxomys whiteheadi* | A | M | 15 | 26 | 116 | 120 | 60 | 20/05/2022 | Marup Atas, Engkilili | SAMN37668514 |
| Mwhit08 | SFR24-052 | *Maxomys whiteheadi* | A | M | 17 | 27 | 112 | 116 | 55 | 25/11/2024 | Sabal Forest Reserve | SAMN48267160 |
| Mwhit09 | SFR24-075 | *Maxomys whiteheadi* | A | F | 14 | 24 | 102 | 84 | 60 | 26/11/2024 | Sabal Forest Reserve | SAMN48267161 |
| Mwhit10 | SFR24-094 | *Maxomys whiteheadi* | A | F | 14 | 25 | 104 | 96 | 50 | 27/11/2024 | Sabal Forest Reserve | SAMN48267162 |
| Mwhit11 | SFR24-113 | *Maxomys whiteheadi* | A | M | 16 | 27 | 110 | 107 | 60 | 28/11/2024 | Sabal Forest Reserve | SAMN48267163 |
| Mwhit12 | SFR24-054 | *Maxomys whiteheadi* | A | M | 16 | 26 | 100 | 102 | 65 | 25/11/2024 | Sabal Forest Reserve | SAMN48267164 |
| Mwhit13 | SFR24-055 | *Maxomys whiteheadi* | A | M | 14 | 28 | 118 | 103 | 70 | 25/11/2024 | Sabal Forest Reserve | SAMN48267165 |
| Mwhit14 | SFR24-057 | *Maxomys whiteheadi* | A | M | 17 | 25 | 111 | 84 | 55 | 25/11/2024 | Sabal Forest Reserve | SAMN48267166 |
| Mwhit15 | SFR24-070 | *Maxomys whiteheadi* | A | M | 12 | 27 | 122 | 100 | 80 | 26/11/2024 | Sabal Forest Reserve | SAMN48267167 |
| Mwhit16 | SFR24-071 | *Maxomys whiteheadi* | A | F | 14 | 24 | 114 | 111 | 60 | 26/11/2024 | Sabal Forest Reserve | SAMN48267168 |
| Ncrem01 | GR24-017 | *Niviventer cremoriventer* | A | M | 17 | 25 | 182 | 99 | 70 | 20/10/2024 | Gua Raya, Serian | SAMN48267169 |
| Ncrem02 | LHNP22-011 | *Niviventer cremoriventer* | A | M | 15 | 27 | 172 | 145 | 70 | 02/07/2022 | Lambir Hills National Park | SAMN48267170 |
| Ncrem03 | LHNP22-014 | *Niviventer cremoriventer* | A | M | 14 | 26 | 170 | 139 | 70 | 03/07/2022 | Lambir Hills National Park | SAMN48267171 |
| Ncrem04 | LHNP22-015 | *Niviventer cremoriventer* | J | M | 14 | 23 | 135 | 101 | 55 | 03/07/2022 | Lambir Hills National Park | SAMN37668510 |
| Ncrem05 | MAE22-005 | *Niviventer cremoriventer* | A | M | 12 | 26 | 177 | 135 | 75 | 20/05/2022 | Marup Atas, Engkilili | SAMN37668512 |
| Ncrem06 | SFR24-072 | *Niviventer cremoriventer* | A | M | 10 | 23 | 179 | 127 | 115 | 26/11/2024 | Sabal Forest Reserve | SAMN48267172 |
| Ncrem07 | SFR24-085 | *Niviventer cremoriventer* | A | F | 12 | 25 | 187 | 129 | 70 | 27/11/2024 | Sabal Forest Reserve | SAMN48267173 |
| Ncrem08 | SFR24-089 | *Niviventer cremoriventer* | J | F | 14 | 24 | 137 | 100 | 40 | 27/11/2024 | Sabal Forest Reserve | SAMN48267174 |
| Ncrem09 | SFR24-056 | *Niviventer cremoriventer* | J | M | 10 | 21 | 127 | 91 | 45 | 25/11/2024 | Sabal Forest Reserve | SAMN48267175 |
| Ncrem10 | SSK22-014 | *Niviventer cremoriventer* | A | F | 16 | 26 | 175 | 133 | 57 | 16/07/2022 | Sungai Sibau, Kapit | SAMN37668519 |
| Ncrem11 | SSK22-017 | *Niviventer cremoriventer* | A | F | 16 | 24 | 165 | 128 | 65 | 17/07/2022 | Sungai Sibau, Kapit | SAMN37668520 |
| Ncrem12 | UPK23-006 | *Niviventer cremoriventer* | A | F | 17 | 25 | 145 | 115 | 70 | 12/03/2023 | Ulu Poi, Kanowit | SAMN37668522 |
| Rtane01 | TAZ24-025 | *Rattus tanezumi* | A | M | 22 | 39 | 204 | 163 | 135 | 13/12/2024 | Tun Ahmad Zaidi Nature Reserve | SAMN48267176 |
| Rtane02 | UPK23-009 | *Rattus tanezumi* | A | M | 12 | 31 | 145 | 110 | 55 | 12/03/2023 | Ulu Poi, Kanowit | SAMN37668523 |
| Rtiom01 | SFR24-068 | *Rattus tiomanicus* | J | F | 10 | 29 | 123 | 85 | 50 | 26/11/2024 | Sabal Forest Reserve | SAMN48267177 |
| Rtiom02 | SFR24-073 | *Rattus tiomanicus* | A | F | 14 | 30 | 145 | 125 | 110 | 26/11/2024 | Sabal Forest Reserve | SAMN48267178 |
| Smuel01 | GR24-001 | *Sundamys muelleri* | A | M | 19 | 45 | 265 | 224 | 310 | 19/10/2024 | Gua Raya, Serian | SAMN48267179 |
| Smuel02 | LHNP22-016 | *Sundamys muelleri* | A | M | 22 | 45 | 263 | 219 | 223 | 03/07/2022 | Lambir Hills National Park | SAMN37668511 |
| Smuel03 | SFR24-065 | *Sundamys muelleri* | J | M | 15 | 30 | 135 | 105 | 55 | 26/11/2024 | Sabal Forest Reserve | SAMN48267180 |
| Smuel04 | SFR24-090 | *Sundamys muelleri* | A | F | 17 | 37 | 232 | 172 | 155 | 27/11/2024 | Sabal Forest Reserve | SAMN48267181 |
| Smuel05 | SFR24-093 | *Sundamys muelleri* | A | M | 22 | 41 | 232 | 194 | 165 | 27/11/2024 | Sabal Forest Reserve | SAMN48267182 |
| Smuel06 | SSK22-006 | *Sundamys muelleri* | A | M | 21 | 43 | 275 | 214 | 250 | 15/07/2022 | Sungai Sibau, Kapit | SAMN37668516 |
| Smuel07 | SSK22-007 | *Sundamys muelleri* | A | M | 22 | 46 | 251 | 219 | 340 | 15/07/2022 | Sungai Sibau, Kapit | SAMN48267183 |
| Smuel08 | SSK22-016 | *Sundamys muelleri* | A | M | 22 | 48 | 277 | 211 | 290 | 17/07/2022 | Sungai Sibau, Kapit | SAMN48267184 |

A = Adult, J = Juvenile, M = Male, F = Female, E = Ear, HF = Hindfoot, TV = Tail-ventral, HB = Head-body, WT = Weight

**Supplementary Figure 1.** Heatmap showing the log-transformed abundance of gastroenteritis associated bacteria detected in individual rodent samples. The rodent sample ID abbreviation corresponds to Supplementary Table 1.


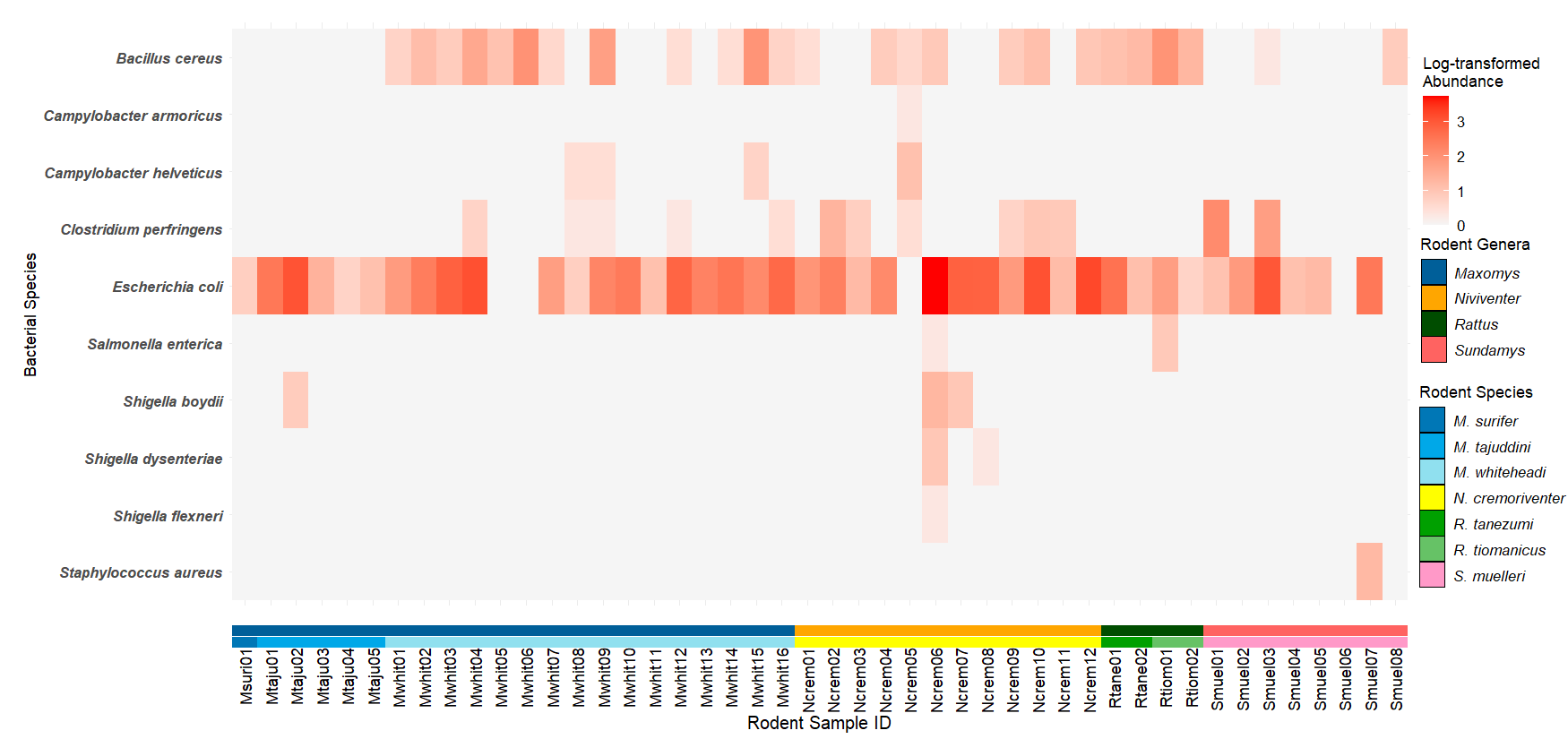

Supplement: Supplementary file 1 — Supplementary Material 1. [file 12866_2025_4241_MOESM1_ESM.docx]
